# Impact of antibiotic use on *Escherichia coli* resistance in goats: A longitudinal cohort study in Selangor, Malaysia

**DOI:** 10.14202/vetworld.2025.2479-2486

**Published:** 2025-08-26

**Authors:** Okti Herawati, Siti Khairani Bejo, Zunita Zakaria, Siti Zubaidah Ramanoon

**Affiliations:** 1 Department of Veterinary Pathology and Microbiology, Faculty of Veterinary Medicine, Universiti Putra Malaysia, 43400, Serdang, Selangor, Malaysia; 2 Department of Farm and Exotic Animal Medicine and Surgery, Faculty of Veterinary Medicine, Universiti Putra Malaysia, 43400, Serdang, Selangor, Malaysia

**Keywords:** antibiotic resistance, antibiotic use, cohort study, *Escherichia coli*, goats, One Health, Selangor

## Abstract

**Background and Aim:**

Antibiotic resistance (ABR) in food animals poses a significant threat to public health under the One Health framework. In Malaysia, *Escherichia coli* is a key indicator organism for antimicrobial resistance (AMR) surveillance. However, limited data exist on the resistance profiles of *E. coli* in goats, particularly in relation to antibiotic usage. This study aimed to evaluate the effect of antibiotic use on the temporal development of ABR in *E. coli* isolated from goat farms in Selangor.

**Materials and Methods:**

A prospective cohort study was conducted on two goat farms: one with a documented history of antibiotic use (Farm 2) and one without (Farm 1). A total of 60 goats (30/farm) were followed for 3 months, with fecal samples collected monthly. *E. coli* isolates were identified and subjected to antimicrobial susceptibility testing using the Kirby–Bauer disk diffusion method. Data were analyzed using Chi-square tests, logistic regression, and Cox proportional hazards modeling.

**Results:**

A significant association was found between antibiotic use and the presence of ABR *E. coli* (odds ratio = 5.82; 95% confidence interval [CI]: 1.12–30.20; p < 0.05). The highest resistance was observed in Farm 2 (96.74%) compared to Farm 1 (57.14%). A hazard ratio of 1.74 (95% CI: 1.03–2.94) indicated increased risk over time. Resistance was detected against critically important human antibiotics, including ciprofloxacin, ampicillin, chloramphenicol, and tetracycline. Notably, resistance to meropenem, an antibiotic not approved for veterinary use, was detected in both farms, suggesting possible environmental or interspecies transmission.

**Conclusion:**

This study confirms that antibiotic use in goat farming significantly influences the development of ABR in *E. coli*. The detection of resistance in farms without antibiotic use underscores the need to investigate other contributing factors, such as environmental residues and horizontal gene transfer. These findings support policy recommendations to restrict antibiotic use in livestock and highlight the urgency for comprehensive AMR surveillance and intervention strategies.

## INTRODUCTION

Antimicrobial resistance (AMR) is a critical global health challenge that affects both human and animal populations [1]. Inadequate or ineffective treatment of bacterial infections can result in persistent infections, ultimately increasing mortality among affected hosts [2]. In goats, AMR has particularly severe consequences, with persistent bacterial infections contributing to mortality rates as high as 86% [2, 3]. Furthermore, AMR negatively impacts productivity, leading to estimated monthly income losses of nearly 11% for goat farmers [4]. In Malaysia, goat production has experienced fluctuations in population size over time [5], and bacterial infections have been implicated in mortality rates of up to 22%, further contributing to the declining goat population [6].

Surveillance of AMR is essential for developing targeted prevention strategies and evaluating the magnitude of resistance threats in animal populations [7]. The World Organization for Animal Health (WOAH) has designated *Escherichia coli* as a key sentinel organism for AMR monitoring due to its widespread presence and ability to acquire and disseminate resistance genes. As such, *E. coli* is routinely monitored in feed, animals, and the environment [8].

*E. coli* is a well-established indicator organism for AMR surveillance because of its genetic adaptability and propensity to transfer resistance elements across bacterial populations [9, 10]. It is considered a significant contributor to the development of resistance among Gram-negative bacteria in livestock [11]. In Malaysia, the AMR Action Plan emphasizes AMR surveillance, public awareness, and research efforts to mitigate the spread of resistance [7]. The Department of Veterinary Services has conducted routine monitoring of AMR in *E. coli* from major livestock species, including cattle, pigs, and poultry [12]. However, surveillance data on AMR in goats remain sparse. A previous study by Mansor *et al*. [13] has reported *E. coli* resistance rates ranging from 27% to 100% in dairy goats. This high level of resistance underscores the growing importance of AMR in the goat sector, which constitutes approximately 20% of Malaysia’s total livestock population [5]. Antibiotic use has been identified as a key driver of resistance in goats [14].

Despite growing concern over AMR in livestock, current surveillance in Malaysia has largely focused on cattle, poultry, and swine, with limited attention to goats. This underrepresentation persists even though goats account for approximately one-fifth of Malaysia’s total livestock population [5]. Most available studies on AMR in goats have concentrated on clinical cases or dairy populations [13, 15], which may not reflect the broader resistance dynamics within healthy, non-diseased goat herds. Moreover, these studies are typically cross-sectional and fail to capture the temporal evolution of resistance following antibiotic exposure. There is also a lack of longitudinal data examining how antibiotic usage in farm settings influences the resistance profile of *E. coli*–a key surveillance organism and reservoir for resistance genes–in goat populations. This presents a significant knowledge gap in understanding the development and drivers of AMR under field conditions, particularly in relation to antibiotic use practices on Malaysian goat farms.

To address this gap, the present study aimed to investigate the impact of antibiotic use on the antibiotic resistance (ABR) profile of *E. coli* isolated from goats in Selangor, Malaysia, through a longitudinal cohort approach. Specifically, this study sought to (i) monitor the development of ABR over time in goat farms with and without a history of antibiotic use, (ii) identify specific antibiotics to which resistance emerges following usage, and (iii) determine the magnitude of association between antibiotic administration and the likelihood of resistant *E. coli* emergence. By capturing resistance patterns at multiple timepoints and comparing farms with different antibiotic use histories, this study provides valuable insights into the temporal dynamics of AMR in goats. The findings are intended to support the implementation of Malaysia’s National Action Plan on AMR and inform prudent antibiotic use policies in small ruminant production systems.

## MATERIALS AND METHODS

### Ethical approval

Ethical clearance was granted by the Institutional Animal Care and Use Committee of Universiti Putra Malaysia (Approval No: UPM/IACUC/AUP-015/2022). This study followed the Animals in Research: Reporting *In vivo* Experiments guidelines.

### Study period and location

This study was conducted from July to October 2023 in Selangor, Malaysia.

### Sample size determination

A prospective cohort study was conducted based on a preliminary cross-sectional assessment involving 450 goats from 27 farms in Selangor, Malaysia. The sample size for the cross-sectional phase was calculated using a standard formula with a Z-score of 1.96, a 5% margin of error (d = 0.05), and an expected prevalence (p) of 50%, yielding a required minimum of 384 goats. The sample size was calculated and increased based on the previous studies [16, 17]. For the cohort phase, assuming a relative risk of 1.9, a baseline incidence of 38% in the unexposed group, 80% power, and 95% confidence, the minimum required sample size was determined to be 60 goats (30 per group), using Epitools [18–20].

### Farm selection criteria

Farms were selected based on uniformity in key management practices to minimize confounding. Selected farms utilized non-pasture systems, limited outsider access, and followed similar feeding regimes. Environmental variables such as antibiotic residue levels, disinfectant use, and metal exposure were not controlled due to the unavailability of baseline data. Two farms were selected from the cross-sectional study: Farm 1 (no antibiotic use) and Farm 2 (routine antibiotic use, as documented in farm records). Both farms previously yielded *E. coli* isolates susceptible to the antibiotics tested, which included ampicillin (AMP), amikacin (AK), gentamicin (CN), amoxicillin/clavulanic acid (AMC), chloramphenicol (C), ciprofloxacin (CIP), nalidixic acid (NA), norfloxacin (NOR), ceftriaxone (CRO), meropenem (MEM), sulfamethoxazole/trimethoprim (SXT), and tetracycline (TE).

### Longitudinal sampling procedure

The study was conducted between July and October 2023. Thirty goats aged approximately 1.5 years were randomly selected per farm and tagged for follow-up. Fecal samples were collected monthly for 3 months (3 timepoints per goat). Samples were obtained aseptically through rectal collection using sterile gloves, stored in sterile containers, and transported in cool boxes (≤4°C) to the laboratory for immediate processing [21]. This sampling frequency was designed to capture temporal shifts in AMR and detect latent or emerging resistance profiles [22–24].

### Isolation and identification of *E. coli*

*E. coli* isolation followed protocols from the Faculty of Veterinary Medicine, Universiti Putra Malaysia, and the WOAH guidelines [25]. Fecal samples were diluted in sterile distilled water and plated on MacConkey agar. Colonies exhibiting typical *E. coli* morphology were subcultured and confirmed through Gram staining and standard biochemical assays, including urease, citrate, oxidase, triple sugar iron, and sulfide indole motility tests [25, 26].

### Antimicrobial susceptibility testing (AST)

AST was performed using the Kirby–Bauer disk diffusion method on Mueller–Hinton agar in accordance with Clinical and Laboratory Standards Institute (CLSI) M100 guidelines (30^th^ edition) [27]. Inocula were standardized to 0.5 McFarland turbidity and tested against 12 antibiotics: AMP, AK, CN, AMC, C, CIP, NA, NOR, CRO, MEM, STX, and TE [27]. Antibiotics were selected based on their relevance to livestock and human medicine and their classification under One Health surveillance priorities [28].

### Statistical analysis

All data analyses were performed using Statistical Package for the Social Sciences version 22 (IMB Corp., NY, USA). Descriptive statistics summarized resistance patterns, and Chi-square tests (p < 0.10) were used to identify potential predictors for multivariate analysis. Logistic regression was applied to determine the adjusted odds ratios (OR) for ABR. In addition, Cox proportional hazards regression assessed time-dependent associations between antibiotic use and resistance emergence, with results presented as hazard ratios and 95% confidence intervals (CIs) [29, 30].

## RESULTS

### Prevalence of ABR *E. coli* (ABREC)

**Figure 1:**
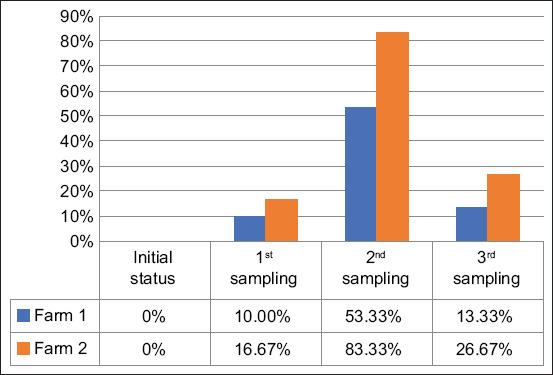
Comparison of antibiotic-resistant *Escherichia coli* levels across three sampling periods. A significant increase in resistance was observed in the 2^nd^ sampling for both farms, with Farm 2 showing the highest resistance (83.33%) (p < 0.05).

*E. coli* isolates exhibiting resistance to at least one of the antibiotics tested were classified as ABREC. At the first sampling point, the prevalence of ABREC was 10% in Farm 1 (antibiotic free) and 16.67% in Farm 2 (antibiotic using). ABREC was consistently detected in subsequent sampling rounds across both farms (Figure 1). Goats in Farm 2 demonstrated a significantly higher risk of harboring ABREC, with a hazard ratio of 1.74 (95% CI: 1.03–2.94), indicating a 74% increased likelihood of developing resistance compared to goats in Farm 1.

Although no statistically significant difference in ABREC prevalence was observed at baseline (p > 0.05), a significant divergence emerged during the second sampling. At this point, 53.33% of goats in Farm 1 and 83.33% in Farm 2 tested positive for ABREC, highlighting a temporal association between antibiotic use and increased resistance levels.

### Temporal changes in resistance profiles

**Figure 2:**
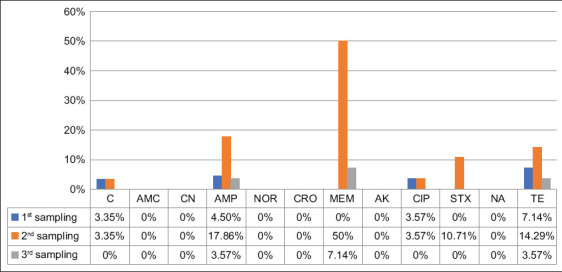
Antibiotic resistance (ABR) profile during the study period in Farm 1. In Farm 1, ABR was observed for C, AMP, CIP, MEM, and TE across the study. C=Chloramphenicol, AMC=Amoxicillin/clavulanic acid, CN=Gentamicin, AMP=Ampicillin, NOR=Norfloxacin, CRO=Ceftriaxone, MEM=Meropenem, AK=Amikacin, CIP=Ciprofloxacin, STX=Sulfamethoxazole, NA=Nalidixic acid, and TE=Tetracycline.

**Figure 3:**
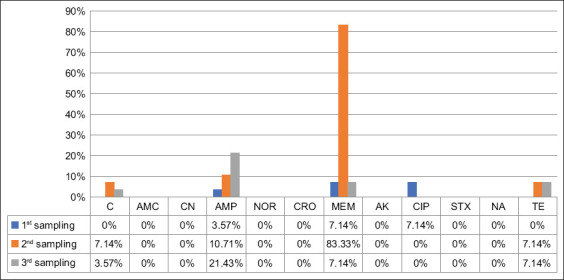
Antibiotic resistance profile during the longitudinal study in Farm 2. Resistance to C, AMP, MEM, and TE was observed throughout the study. C=Chloramphenicol, AMC=Amoxicillin/clavulanic acid, CN=Gentamicin, AMP=Ampicillin, NOR=Norfloxacin, CRO=Ceftriaxone, MEM=Meropenem, AK=Amikacin, CIP=Ciprofloxacin, STX=Sulfamethoxazole, NA=Nalidixic acid, and TE=Tetracycline.

AST revealed evolving resistance patterns during the study. In Farm 1, resistance developed against CIP, MEM, AMP, TE, STX, and C (Figure 2). Farm 2 exhibited resistance to all of these antibiotics except STX (Figure 3). Notably, all goats across both farms remained consistently susceptible to CN, AMC, CRO, NOR, AK, and NA throughout the 3-month observation period.

### Risk factors associated with ABREC occurrence

Out of 60 goats sampled over the study period, 46 (76.67%) tested positive for ABREC—19 (63.33%) from Farm 1 and 27 (90%) from Farm 2. Univariate analysis showed that antibiotic use was significantly associated with ABREC (χ² = 5.96, p = 0.015) (Table 1). In addition, body cleanliness was also found to be significantly associated with ABREC occurrence (χ^2^ = 4.10, p = 0.043). Goats from antibiotic-using farms were approximately 5 times more likely to harbor ABREC (OR = 5.21; 95% CI: 1.28–21.24).

**Table 1:** Univariate analysis of the risk factors associated with ABREC in goats in Selangor.

Variable	Number of samples	Positive ABREC (%)	Chi-square (χ^2^)	p-value	OR (95% CI)
Antibiotic use					
Yes	30	90	5.96	0.015*	5.21 (1.28–21.24)
No	30	63.33		Ref.	
Health status					
Healthy, Yes	57	77.19	0.18	0.674	0.59 (0.50–7.049)
Sick, No	3	66.67		Ref.	
Body cleanliness					
Dirty	11	100	4.10	0.043*	1.40 (1.17–1.67)
Clean	49	71.43		Ref.	

* Statistically significant (p < 0.05), OR=Odds ratio, Ref.=Reference, ABREC=Antibiotic-resistant *Escherichia coli*

### Multivariate analysis of risk factors

Multivariable logistic regression further confirmed antibiotic use as a significant predictor of ABREC occurrence (OR = 5.82; 95% CI: 1.12–30.20; p < 0.05). In contrast, body hygiene was not significantly associated with ABREC presence after adjusting for other variables (p > 0.05), suggesting that antibiotic use was the dominant factor influencing resistance emergence (Table 2).

**Table 2:** Results of the multivariate logistic regression analysis of the risk factors associated with ABREC development in goats in Selangor.

Variable	Coefficient	SE	Chi-square (χ^2^)	p-value	OR (95% CI)
Intercept	39.14	1.39	0.078	0.999	
Antibiotic use					
Yes	1.76	0.84	4.40	0.036*	5.82 (1.12–30.20)
No				Ref.	
Body cleanliness					
Dirty	− 38.70	1.39	0.077	0.999	0.015 (0.000 – 1.000)
Clean				Ref.	

* Statistically significant (p < 0.05), ABREC=Antibiotic-resistant *Escherichia coli*, SE=Standard error, OR=Odds ratio, CI=Confidence interval, Ref.=Reference

## DISCUSSION

### Antibiotic use and time-dependent resistance development

This study demonstrated a significant relationship between antibiotic use in goats and the development of ABR over time. A hazard ratio of 1.74 indicated that goats on antibiotic-using farms had a 74% higher risk of developing *E. coli* resistance compared to goats on farms that did not administer antibiotics. These findings align with previous reports that antibiotic exposure accelerates the selection and spread of resistant bacteria in livestock populations [31, 32]. Antibiotics can induce resistance through multiple mechanisms, including porin modification, mutagenesis, and oxidative stress-related DNA damage [33, 34]. The selective pressure exerted by subtherapeutic antibiotic concentrations also favors the survival of resistant strains, contributing to resistance propagation across microbial communities [34–37].

### Resistance emergence timeline and the role of farm stressors

The onset of *E. coli* resistance was evident from the 1^st^ month of sampling, suggesting rapid microbial adaptation following antibiotic exposure. While literature suggests that AMR emergence can occur within 1–6 months post-exposure, the timeline remains poorly defined and may vary based on antibiotic type, dosage, and host-microbiome dynamics [38, 39]. Farm-related stressors such as biocides, pesticides, and heavy metals further contribute to resistance selection by inducing bacterial stress responses that promote mutagenesis and gene transfer [40–42]. Our findings support the hypothesis that a greater number and diversity of stressors exacerbate resistance development. Notably, ABR may be reversible upon the removal of stressors, as prior studies have shown a reduction in resistance within weeks after eliminating selective pressures [43].

### Resistance to critically important antibiotics

The study revealed that *E. coli* isolates exhibited resistance to multiple critically and highly important antibiotics for human medicine, including CIP, AMP, C, TE, and STX [44]. These antibiotics are frequently used in Malaysian livestock systems and have been detected as residues in terrestrial and aquatic farm environments [45–47]. The widespread detection of resistance, even in farms without documented antibiotic use, points to the potential role of environmental contamination and indirect antibiotic exposure in resistance development [48].

### Unexpected detection of MEM resistance

An unexpected and concerning finding was the detection of MEM-resistant *E. coli* on both farms. MEM, a last-resort carbapenem antibiotic not approved for veterinary use, is typically reserved for treating multidrug-resistant infections in humans [45, 49, 50]. The detection of resistance to such a drug in goats suggests environmental or horizontal gene transfer mechanisms, possibly through plasmid-mediated resistance, contaminated feed, or co-resistance driven by disinfectant use [51]. Both farms reported regular use of disinfectants, which can promote co-selection of resistance genes through mechanisms such as co-resistance, cross-resistance, and co-regulation [40]. These findings underscore the need for further molecular investigation into the origin and drivers of carbapenem resistance in livestock settings.

### Second sampling and resistance surge

A pronounced increase in resistance, particularly to MEM, was observed during the second sampling period. This surge may be explained by cumulative exposure to stressors or increased selective pressure over time [52]. Elevated resistance levels correlate with higher inducer concentrations, including antibiotics, heavy metals, and biocides, which trigger cellular responses such as efflux pump activation, increased mutation rates, and horizontal gene transfer events [33, 34, 41].

### Resistance in antibiotic-free farms and environmental transmission

Interestingly, STX-resistant *E. coli* was isolated from goats in the antibiotic-free farm. Previous studies by Thiang *et al*. [46] and Marni *et al*. [47] have reported widespread antibiotic residues, including TE, sulfonamides, and quinolones, in livestock and aquaculture environments in Malaysia. The persistence of these residues in soil and water can contribute to resistance, even in the absence of direct antibiotic use. In addition, non-antibiotic selective agents such as biocides and metal residues can facilitate resistance through indirect selection pathways [40]. The spread of resistance genes from wildlife, environmental sources, and human activity further complicates containment efforts [53, 54]. These findings emphasize the importance of monitoring not only antibiotic usage but also environmental contaminants and interspecies transmission routes.

## CONCLUSION

This longitudinal cohort study demonstrated a significant association between antibiotic use and the emergence of ABREC in goats. Goats raised on farms with antibiotic use exhibited a higher prevalence of ABREC (90%) compared to those on antibiotic-free farms (63.33%), with a hazard ratio of 1.74, indicating a 74% increased risk of developing resistance over time. Resistance was detected against several critically important antibiotics for human medicine, including CIP, AMP, TE, C, and STX. Importantly, resistance to MEM–an antibiotic not approved for veterinary use–was observed on both farms, suggesting the potential role of environmental exposure or horizontal gene transfer in resistance development.

The findings have important practical implications for antimicrobial stewardship in livestock systems. Limiting antibiotic use in goat farming may help suppress resistance emergence, and surveillance programs should be expanded to include small ruminants under the Malaysian AMR Action Plan. The consistent detection of resistance even in farms that did not use antibiotics highlights the need for broader control measures, including improved farm hygiene, controlled use of biocides and disinfectants, and environmental monitoring for antibiotic residues.

A key strength of this study is its prospective cohort design, which enabled the capture of temporal changes in resistance profiles and strengthened causal inference. Standardized sampling procedures and well-matched farm conditions reduced potential confounding. However, the study is limited by the absence of molecular characterization of resistance genes and the lack of quantification of environmental residues such as antibiotics, metals, or disinfectants. These gaps restrict the ability to fully determine the sources and mechanisms driving resistance.

Future studies should incorporate genomic analyses of resistant isolates, evaluate antibiotic and biocide residues in farm environments, and track the dissemination pathways of resistance genes. Expanding the geographic and production scope of AMR surveillance in goats will further strengthen evidence-based policy development.

In conclusion, this study underscores the role of antibiotic use in driving AMR in *E. coli* from goats and highlights the importance of addressing indirect transmission pathways involving environmental and interspecies factors. These insights reinforce the necessity of a One Health approach to AMR mitigation, promoting sustainable livestock production while safeguarding public health.

## AUTHORS’ CONTRIBUTIONS

OH, SKB, ZZ, and SZR: Designed the study. OH and SKB: Collected the samples. OH: Analyzed the samples and wrote and edited the manuscript. OH and SZR: Performed the statistical analysis. All authors have read and approved the final version of the manuscript.

## References

[1] WHO (World Health Organization) (2023). Incentivizing the Development of New Antibacterial Treatments Progress Report.

[2] Andersson M, Ostholm-Balkhed A, Fredrikson M, Holmbom M, Hallgren A, Berg S, Hanberger H (2019). Delay of appropriate antibiotic treatment is associated with high mortality in patients with community-onset sepsis in a Swedish setting. Eur. J. Clin. Microbiol. Infect. Dis.

[3] Pagwesese D.P, Made J, Muchinguri-Kashiri O (2017). Situational Analysis of Antimicrobial use and Resistance in Humans and Animals in Zimbabwe. The Center for Disease Dynamics, Economics and Policy, Washington.

[4] Martins S.B, Afonso J.S, Fastl C, Huntington B, Rushton J (2024). The burden of antimicrobial resistance in livestock:A framework to estimate its impact within the global burden of animal diseases programme. One Health.

[5] DVS (Department of Veterinary Services) (2022). Livestock Statistics 2023/2024. Jabatan Perkhidmatan Veterinar Malaysia, Putrajaya.

[6] Mahmood Z.K.H, Jesse F.F, Saharee A.A, Jasni S, Yusoff R, Wahid H (2015). Clinio-pathological changes in goats challenged with. Corynebacterium peudotuberculosis and its exotoxin (PLD). *Am. J. Anim. Vet. Sci*.

[7] MOH (Ministry of Health) (2022). Malaysian Action Plan on Antimicrobial Resistance (MyAP-AMR) 2022-2026. Attin Press, Putrajaya.

[8] WOAH (World Organization for Animal Health) (2020). Standards, Guidelines and Resolutions on Antimicrobial Resistance and The Use of Antimicrobial Agents.

[9] Anjum M.F, Schmitt H, Bo¨Rjesson S, Berendonk T.U, Stehling E.G, Boerlin P, Topp E, Jardine C, Li X, Li B, Dolejska M, Madec J.Y, Dagot C, Guenther S, Walsh F, Villa L, Veldman K, Sunde M, Krzeminski P, Wasyl D, Popowska M, Ja¨Rhult J, Stefan O, Mahjoub O, Mansour W, Tha´I D.N, Elving J, Pedersen K, Behalf of the WAWES Network Erica Donner (2021). The potential of using. E. Coli as an indicator for the surveillance of antimicrobial resistance (AMR) in the environment. Curr. Opin. Microbiol.

[10] Sali V, Nykasenoja S, Heikinheimo A, Halli O, Trikkonen T, Heinonen M (2021). Antimicrobial use and susceptibility of indicator. Escherichia coli in Finnish integrated pork production. *Front. Microbiol*.

[11] EFSA (European Food Safety Authority) (2012). Technical specifications on the harmonised monitoring and reporting of antimicrobial resistance in. Salmonella, *Campylobacter* and indicator *Escherichia coli* and *Enterococcus* spp. *Bacteria* transmitted through food. *EFSA J*.

[12] DVS (2021). Data Survelan Antimicrobial Resistance (AMR) Kebangsaan 2018 - 2021.

[13] Mansor R, Diauudin N.S, Syed-Hussain S.S, Khalid S.F (2019). Antibiotic susceptibility of Staphylococcus aureus and *Escherichia coli* isolated from dairy goats in selected farms in Selangor, Malaysia.. J. Vet. Malaysia.

[14] Alhaji N.B, Haruna A.E, Isola T.O, Odetokun I.A (2021). Antimicrobial usage and resistance in small ruminant food animals in Nigeria:Drivers for misuse, pathways for dissemination and public health impacts. Int. J. Infect. Dis.

[15] Haulisah N.A, Hassan L, Bejo S.K, Jajere S.M, Ahmad N.I (2021). High levels of antibiotic resistance in isolates from diseased livestock. Front. Vet. Sci.

[16] Daniel W.W, Cross C.L (1991). Biostatistics:A Foundation for Analysis in the Health Sciences.

[17] Memon M.A, Ting H, Cheah J.H, Thurasamy R, Chuah F, Cham T.H (2020). Sample size for survey research:Review and recommendations. J. Appl. Struct. Equ. Model.

[18] Chandrasekaran S, Sreedharan J, Gopakumar A (2020). Sample size estimation in cohort studies for testing of relative risk. Int. J. Sci. Technol. Res.

[19] Elmi S.A, Simons D, Elton L, Haider N, Abdel Hamid M.M, Shuaib Y.A, Khan M.A, Othman I, Kock R, Osman A (2021). Identification of risk factors associated with resistant. Escherichia coli isolates from poultry farms in the east coast of peninsular Malaysia:A cross sectional study. Antibiotics (Basel).

[20] Rahmahani J, Mufasirin M, Tyasningsih W, Effendi M.H (2020). Antimicrobial resistance profile of Escherichia coli from cloacal swab of domestic chicken in Surabaya traditional market. Biochem. Cell. Arch.

[21] Jahan S, Purkayastha M, Elahi A.T.M.M, Rahman M.L, Choudhury S, Kahir M.A, Das M (2021). Isolation, identification and antibiogram study of bacteria from diarrhoeic goat at selected areas of Sylhet. Int. J. Nat. Sci.

[22] WHO (2013). Integrated Surveillance of Antimicrobial Resistance Guidance from a WHO Advisory Group.

[23] Ndegwa E, Almehmadi H, Chyer K, Kaseloo P, Ako A.A (2019). Longitudinal shedding patterns and characterization of antibiotic resistant. E. Coli in pastured goats using a cohort study. *Antibiotics (Basel)*.

[24] Deekshit V.K, Srikumar S (2022). 'To be, or not to be'-the dilemma of 'silent'antimicrobial resistance genes in. Bacteria. *J. Appl. Microbiol*.

[25] WOAH (2018). Verotoxigenic. Escherichia coli. World Organization for Animal Health, Paris.

[26] Jang S.S, Biberstein E.L, Hirsh D.C (2008). A Diagnostic Manual of Veterinary Clinical Bacteriology and Mycology.

[27] CLSI (Clinical and Laboratory Standards Institute) (2023). Performance Standards for Antimicrobial Susceptibility Testing.

[28] WHO (2024). List of Medically Important Antimicrobials:A Risk Management Tool for Mitigating Antimicrobial Resistance due to Non-human Use.

[29] Thiese M.S, Ronna B, Ott U (2016). P value interpretations and considerations. J. Thorac. Dis.

[30] Malhotra R.K (2020). Errors in the use of multivariable logistic regression analysis:An empirical analysis. Indian J. Community Med.

[31] Samreen Ahmad, I, Malak H.A, Abulreesh H.H (2021). Environmental antimicrobial resistance and its drivers:A potential threat to public health. J. Glob. Antimicrob. Resist.

[32] Munos-Price L.S, Frencken J.F, Tarima S, Bonten M (2016). Handling time-dependent variables:Antibiotics and antibiotic resistance. Clin. Infect. Dis.

[33] Zhang F, Cheng W (2022). The mechanism of bacterial resistance and potential bacteriostatic strategies. Antibiotics (Basel).

[34] Revitt-Mills S.A, Robinson A (2020). Antibiotic-induced mutagenesis:Under the microscope. Front. *Microbiol*.

[35] Huong L.Q, Thuy N.T.B, Anh N.T.L, Thuy D.T.T, Thanh D.T.H, Padungto P (2021). Antibiotics use in fish and shrimp farms in Vietnam. Aquac. Rep.

[36] Smith R.P, May H.E, Abuoun M, Stubberfield E, Gilson D, Chau K.K, Crook D.W, Shaw L.P, Read D.S, Stoesser N, Vilar M.J, Anjum M.F (2023). A longitudinal study reveals persistence of antimicrobial resistance on livestock farms is not due to antimicrobial usage alone. Front. Microbiol.

[37] Ruiz J, Gordon M, Villarreal E, Frasquet J, Sanchez M.A, Martin M, Castellanos A, Ramirez P (2019). Influence of antibiotic pressure on multidrug-resistant. Klebsiella pneumoniae colonisation in critically ill patients. *Antimicrob. Resist. Infect*
*Control*.

[38] Poku E, Cooper K, Cantrell A, Harnan S, Sin M.A, Zanuzdana A, Hoffmann A (2023). Systematic review of time lag between antibiotic use and rise of resistant pathogens among hospitalized adults in Europe. JAC Antimicrob. Resist.

[39] Sulaiman J.E, Lam H (2021). Evolution of bacterial tolerance under antibiotic treatment and its implications on the development of resistance. Front. Microbiol.

[40] GBD 2021 Antimicrobial Resistance Collaborators (2024). Global burden of bacterial antimicrobial resistance 1990-2021:A systematic analysis with forecasts to 2050. Lancet.

[41] Dawan J, Ahn J (2022). Bacterial stress responses as potential targets in overcoming antibiotic resistance. Microorganisms,.

[42] Poole K (2005). Efflux-mediated antimicrobial resistance. J. Antimicrob. Chemother.

[43] Andersson D.I, Hughes D (2011). Persistence of antibiotic resistance in bacterial populations. FEMS Microbiol. Rev.

[44] WHO (2021). WHO Antimicrobial Resistance. Availablefrom:https://www.nichd.nih.gov/health/topics/preterm/conditioninfo/who-risk.

[45] DVS (2021). Malaysian Veterinary Antimicrobial Guidelines (MVAG).

[46] Thiang E.L, Leea C.W, Takada H, Seki K, Takei A, Wang S.S.A, Bong C.W (2021). Antibiotic residues from aquaculture farms and their ecological risks in Southeast Asia:A case study from Malaysia. Ecosyst. Health Sustain.

[47] Marni S, Marzura M.R, Eddy A.A, Suliana A.K (2017). Veterinary drug residues in chicken, pork and beef in peninsular Malaysia in the period 2010-2016. Malays. J. Vet. Res.

[48] Sorn S, Sulfikar Lin M.Y, Shuto M, Noguchi M, Honda R, Yamamoto-Ikemoto R, Watanabe T (2022). Potential impact factors on the enhancement of antibiotic resistance in a lake environment. J. Water Health.

[49] Ramirez-Castillo F.Y, Guerrero-Barrera A.L, Avelar-Gonzalez F.J (2023). An overview of carbapenem-resistant organisms from food-producing animals, seafood, aquaculture, companion animals, and wildlife. Front. Vet. Sci.

[50] Teo S.W, Ong H.C, Ng R.X, Kukreja A, Johari B.M, Basri S, Lee C.E, Ponnampalavanar S (2023). Assessing the appropriate use of meropenem and the association of acceptance of antimicrobial stewardship (Ams) interventions with adverse outcomes in a Malaysia tertiary hospital. Int. J. Infect. Dis.

[51] Huang E, Yang X, Leighton E, Li X (2023). Carbapenem resistance in the food supply chain. J. Food Prot.

[52] Olesen S.W, Barnett M.L, Macfadden D.R, Brownstein J.S, Hernandez-Diaz S, Lipsitch M, Grad Y.H (2018). The distribution of antibiotic use and its association with antibiotic resistance. Elife.

[53] Mitchell J (2023). Antimicrobial resistance (AMR) as a form of human-wildlife conflict:Why and how nondomesticated species should be incorporated into AMR guidance. Ecol. Evol.

[54] Zhu Y, Wang C, Li F (2015). Impact of dietary fiber/starch ratio in shaping caecal microbiota in rabbits. Can. J. Microbiol.

